# Association of a Shortened Duration of Adjuvant Chemotherapy With Overall Survival Among Individuals With Stage III Colon Cancer

**DOI:** 10.1001/jamanetworkopen.2021.3587

**Published:** 2021-03-30

**Authors:** Devon J. Boyne, Winson Y. Cheung, Robert J. Hilsden, Tolulope T. Sajobi, Atul Batra, Christine M. Friedenreich, Darren R. Brenner

**Affiliations:** 1 Oncology Outcomes Initiative, University of Calgary, Calgary, Alberta, Canada; 2 Department of Oncology, Cumming School of Medicine, University of Calgary, Calgary, Alberta, Canada; 3 Department of Community Health Sciences, Cumming School of Medicine, University of Calgary, Calgary, Alberta, Canada; 4 Department of Cancer Epidemiology and Prevention Research, Cancer Care Alberta, Alberta Health Services, Calgary, Alberta, Canada; 5 Department of Medicine, Cumming School of Medicine, University of Calgary, Calgary, Alberta, Canada

## Abstract

**Importance:**

Several real-world oncology studies have produced findings that contradict those from randomized clinical trials. Such disparities may be associated with methodological shortcomings.

**Objective:**

To examine the association between a shortened duration of adjuvant chemotherapy among individuals with stage III colon cancer using real-world data.

**Design, Setting, and Participants:**

This comparative effectiveness study included individuals diagnosed with stage III colon cancer between January 2004 and December 2015 who initiated adjuvant chemotherapy at oncology clinics within the province of Alberta, Canada. Patients were identified through record linkage of various administrative databases and were followed up until September 2017. Eligibility criteria were modeled after those used in the International Duration Evaluation of Adjuvant (IDEA) trial. A target trial emulation and naive observational analysis were conducted. Results from both cohorts were benchmarked against findings from the IDEA trial. Data analysis was conducted from March to December 2020.

**Exposure:**

A shortened duration of adjuvant 5-fluorouracil/leucovorin plus oxaliplatin (FOLFOX) or capecitabine plus oxaliplatin (CAPOX) chemotherapy, defined as 3 to 5 months of treatment vs 6 months.

**Main Outcomes and Measures:**

Overall survival assessed via vital statistics. The per-protocol hazard ratio (HR) was estimated using a weighted pooled logistic regression model. Subgroup analyses were conducted by treatment regimen (ie, FOLFOX vs CAPOX) and cancer stage (ie, T1-3 and N1 vs T4 or N2).

**Results:**

From an initial cohort of 3086 patients, 485 (16%) were eligible for inclusion in the target trial analysis. The median age was 59 years (range, 19-81 years), and 230 (47%) were women. The maximum follow-up was 11.6 years. Median overall survival was not reached. A total of 90 patients (19%) died. The 5-year Kaplan Meier overall survival estimate was 0.79 (95% CI, 0.75-0.84). Estimates from the trial emulation were similar to those from the IDEA trial. For example, a shortened duration of adjuvant chemotherapy was not associated with overall survival among patients prescribed CAPOX in the IDEA trial (HR, 0.96; 95% CI, 0.85-1.08) or in the trial emulation (HR, 0.96; 95% CI, 0.43-2.14). In contrast, the naive observational analysis suggested that a shortened duration of CAPOX was significantly associated with worse survival (HR, 3.33; 95% CI, 1.04-10.65).

**Conclusions and Relevance:**

In this study, the explicit emulation of a target trial better approximated results from an analogous well-conducted randomized clinical trial.

## Key Points

**Question:**

Are disparities between findings of observational studies and randomized clinical trials regarding the association between a shortened duration of adjuvant chemotherapy and overall survival among individuals with stage III colon cancer attributable to methodological issues?

**Findings:**

This comparative effectiveness study of 485 individuals based on the explicit emulation of a target trial resulted in estimates that were similar in magnitude to those of a well-conducted randomized clinical trial. In contrast, estimates from a routine observational analysis conducted on the same data set were meaningfully different.

**Meaning:**

These findings suggest that the explicit emulation of a target trial can better approximate findings from an analogous randomized clinical trial when conducting comparative efficacy research using real-world data.

## Teaser

This comparative effectiveness study examines the association between a shortened duration of adjuvant chemotherapy among individuals with stage III colon cancer using real-world data.

## Introduction

Since 2005, the standard of care for stage III colon cancer has been surgery followed by 6 months of adjuvant 5-flourouracil/leucovorin plus oxaliplatin (FOLFOX) or capecitabine plus oxaliplatin (CAPOX).^1,2^ In 2018, clinical practice guidelines were changed in response to findings from the International Duration Evaluation of Adjuvant (IDEA) trial, a pooled analysis of 6 randomized clinical trials examining the noninferiority of 3 months of adjuvant chemotherapy vs 6 months.^3,4,5,6,7^ In the primary analysis, the upper limit of the 95% CI for the disease-free survival hazard ratio (HR) exceeded the prespecified statistical noninferiority threshold of 1.12 (HR, 1.07; 95% CI, 1.00-1.15).^4^ However, in subgroup analyses, a shortened duration of adjuvant chemotherapy was noninferior among patients with T1 to T3 and N1 stage disease and among patients prescribed CAPOX.^4^

The findings from the IDEA trial have been controversial within the oncology community.^3,7,8^ For example, a recent worldwide survey of 145 medical oncologists found that approximately 1 in 3 clinicians supported 6 months of adjuvant chemotherapy as the standard of care.^9^ One methodological issue with the IDEA trial was its reliance on an intention-to-treat analysis, which was problematic because rates of adherence were heterogeneous between treatment groups and trials.^8^ While a per-protocol analysis was conducted, the investigators did not adjust for postrandomization variables that could affect adherence.^10^ In addition, the extent to which the trial findings are generalizable to real-world cancer populations may be problematic. For example, Batra et al^11^ found that 59% of individuals with stage II or III colon cancer in a real-world setting would not be eligible for inclusion in a clinical trial.

While comparative efficacy research from real-world settings may help to address such controversies, 2 recent investigations^12,13^ have reported that the findings from routine observational analyses often lead to disparate conclusions when compared with analogous randomized clinical trials. We speculated that such disparity may be attributable to flaws in the design and analysis of real-world data. Specifically, a growing body of literature has shown how observational data can replicate results from an analogous randomized clinical trials through the explicit emulation of a target trial.^14,15^ This approach has been successfully used within various settings, including the prevention of coronary heart disease,^16,17^ detection and prevention of colorectal cancer,^18,19^ safety of hormone therapy,^20^ management of anemia within end-stage kidney disease,^21^ timing of antiretroviral therapy initiation among individuals with HIV,^22^ association of statin use with survival among individuals with cancer,^23^ and efficacy of systemic therapy in colon and pancreatic cancer.^24^ Current real-world evidence surrounding the optimal duration of adjuvant chemotherapy among individuals with stage III colon cancer is limited.^25^ In a previous systematic review,^25^ we found that observational studies investigating this issue have had biases that could be avoided through the explicit emulation of a target trial and that several of these studies reported findings that contradict those of the IDEA trial.

Given limitations of the observational research to date and the controversy surrounding the IDEA trial findings, we examined the association between a shortened duration of adjuvant chemotherapy among individuals diagnosed with stage III colon cancer using real-world data. In addressing this objective, the results from a target trial emulation were compared with a naive observational analysis that did not explicitly emulate a target trial. Results from both observational analyses were benchmarked against those from the IDEA trial.^4,26^

## Methods

This study was designed in accordance with the trial emulation approach described by Hernán and Robins.^14^ The reporting of this study adheres to the Strengthening the Reporting of Observational Studies in Epidemiology (STROBE) reporting guideline^27^ and the International Society for Pharmacoeconomics and Outcomes Research (ISPOR) reporting guideline for nonrandomized studies of secondary data sources. This study was approved by the Health Research Ethics Board of Alberta Cancer Committee. Data were deidentified prior to analysis. Informed consent was waived by the ethics committee because this was an analysis of an existing administrative database in which there was no direct contact with patients.

### Data Sources

The cohort consisted of individuals with stage III colon cancer who were diagnosed in Alberta between 2004 and 2015 and prescribed an adjuvant chemotherapy regimen containing oxaliplatin. A data set was derived through record linkage of various provincial administrative databases via the Oncology Outcomes research initiative. Electronic medical and systemic pharmacy records were used to obtain information on patient, tumor, and treatment characteristics, including the time and dose of each administered drug within each chemotherapy cycle. The hospital discharge abstract database and the national ambulatory care reporting system database were used to identify the presence of comorbid conditions and treatment-related toxic effects using *International Statistical Classification of Diseases, Tenth Revision, Clinical Modification *(*ICD-10-CM*) codes, which are listed in eTable 1 and eTable 2 in the Supplement. Overall survival was ascertained using vital statistics.

### Eligibility Criteria

The eligibility criteria were modeled after those used within the IDEA trial (Table 1). They included having histologically confirmed stage III colon cancer, being aged 18 years or older, and having undergone surgery for colon cancer.

**Table 1.** Eligibility Criteria for Target Trial, Based on IDEA Trial Eligibility Criteria, and Those Used in the Current Study

Target trial eligibility, based on IDEA trial	Criteria used in target trial emulation
Inclusion criteria	
Histologically confirmed AJCC stage III colon cancer	Same
Age ≥18 y	Same
Undergone surgery for colon cancer	Same
Curative surgery no less than 3 and no more than 8 wk prior to randomization and chemotherapy started within 2 weeks after randomization	Receipt of adjuvant chemotherapy within 10 wk of surgery
ECOG PS, 0, 1, or 2^a^	Not hospitalized for more than 14 d in total within the year prior to diagnosis
Signed written informed consent	Not emulated
Exclusion criteria	
Macroscopic or microscopic evidence of residual tumor, ie, R1 or R0 resections	Not emulated; data unavailable
Previous cancer within the last 5 y other than curatively treated basal cell carcinoma of the skin or in situ carcinoma of the cervix	Same
Previous abdomino-pelvic radiotherapy	Same
Current or recent (ie, within 28 d prior to randomization) treatment with another investigational drug or participation in another investigational study	Receipt of adjuvant therapy other than FOLFOX or CAPOX
History or presence of condition that would contraindicate use of investigational drug or place patient at high risk of treatment complications (eg, a known allergy or hypersensitivity to component of treatment)	No initiation of adjuvant chemotherapy within 180 d of surgery
Pregnancy or lactation or child-bearing potential and unwilling to use contraception	No initiation of adjuvant chemotherapy within 180 d of surgery
Pretreatment blood work within acceptable ranges	No initiation of adjuvant chemotherapy within 180 d of surgery
History of clinically relevant psychiatric disability, precluding informed consent	Same
Clinically relevant cardiovascular disease within past 12 mo	Same
History of interstitial lung disease	Same

Abbreviations: AJCC, American Joint Committee on Cancer; ECOG PS, Eastern Cooperative Oncology Group performance status; IDEA, International Duration Evaluation of Adjuvant.

^a^ Some of the trials within the IDEA collaboration were restricted to patients with an ECOG PS of 0 or 1.

### Treatment Strategies and Contrast of Interest

We had planned to compare the following 4 treatment strategies: 3, 4, 5, and 6 months of adjuvant CAPOX or FOLFOX therapy. However, because of the limited sample size of our data set, the shortened treatment duration strategies were combined into a single grouping. Therefore, we compared 3 to 5 months of adjuvant CAPOX or FOLFOX chemotherapy with a 6-month duration. Although different from the treatment group examined in the IDEA trial, this strategy corresponds to National Comprehensive Cancer Network guidelines of 3 to 6 months of adjuvant chemotherapy for certain patients and aligns with how the IDEA trial findings are implemented in some clinical practices.^3^

We estimated the per-protocol effect size comparing the 2 treatment strategies of interest under perfect adherence. As in the IDEA trial, subgroup analyses were conducted by treatment regimen (CAPOX vs FOLFOX) and cancer stage (T1-3 and N1 vs T4 or N2).^4^ It should be noted that the intention-to-treat effect was not estimable given that clinicians would have not intended to treat patients with shorter durations during the time of the analyses.

In our analyses, treatment modifications that were permissible under the IDEA trial protocol were emulated.^28^ Specifically, dose reductions of as much as 50% of the initial dose of the backbone therapy and dose delays of as long as 4 weeks were permissible within our hypothetical target trial to allow for recovery from toxic effects. Temporary omissions, dose reductions of any amount, or a complete cessation of oxaliplatin were also permitted, as was switching from CAPOX to FOLFOX or from a combination therapy to a monotherapy. Patients were allowed to discontinue chemotherapy if there was evidence of clinically significant cardiac toxic effects, acute pancreatitis, kidney failure, or mental illness or if there were 2 or more hospitalizations or emergency medical visits for a treatment-related toxic effect. A list of the *ICD-10-CM* codes used to define the treatment-related toxic effects are described in eTable 2 in the Supplement.

### Outcome, Analysis Time Zero, and Follow-up Period

The outcome of interest was overall survival. Analysis time zero was defined as the time of treatment initiation. Individuals were followed up until death or until administrative censoring in September 2017, whichever occurred first.

### Assignment Procedures

Based on the results of a previous investigation and the expert opinion of a medical oncologist,^29^ the following baseline variables were identified as potential confounders that could affect treatment discontinuation: age at diagnosis (years), sex (male vs female), performance status (the number of days spent in hospital within the year prior to diagnosis was used as a surrogate for performance status), body mass index (calculated as weight in kilograms divided by height in meters squared), Charlson Comorbidity Index (0, 1, or ≥2; determined using the algorithm by Quan et al^30^), distance from home to treatment facility (kilometers), location of residence (urban or rural), tumor and nodal stage (T1-3 and N1 vs T4 or N2), tumor sidedness (left vs right), tumor grade (high vs low), treatment facility (Calgary, Edmonton, or regional/community), period of diagnosis (years), and chemotherapy regimen (FOLFOX or CAPOX). Time-varying covariates related to treatment history were the dose of the backbone agent and oxaliplatin in the last cycle (ie, percentage of the baseline dose) and whether patients switched treatment regimens (yes vs no). Toxic effects were conceptualized as a time-varying confounder and was modeled using 2 variables: prior cardiovascular toxic effect (yes vs no) and the number of noncardiac treatment-related toxic effects (0, 1, or ≥2). A directed acyclic graph describing the assumed structure of time-varying confounding is in eFigure in the Supplement.

### Statistical Analysis 

#### Analysis Plan

The treatment strategies were compared using a 3-stage method.^22,31,32^ An introduction to this method is provided in Hernán,^32^ and further details can be found in Cain^22^ and Chakraborty and Moodie.^31^ First, participants were duplicated in the data set, and a copy of each participant was assigned to each treatment strategy. This step was used to address the issue of unknown chemotherapy duration at baseline. Second, copies were artificially censored when they deviated from the assigned treatment strategy. For example, copies that were assigned to the 3- to 5-month strategy were artificially censored if and when they received more than 5 months of chemotherapy. Similarly, copies were artificially censored if they discontinued chemotherapy earlier than their assigned duration and there was no evidence of toxic effects that would warrant a premature cessation of therapy. Lastly, time-varying inverse probability of censoring weights (IPCWs) were used to address selection bias due to informative artificial censoring.^22,31,32^ The IPCWs represent the inverse probability that a copy remains uncensored at each point, conditional on the baseline and time-varying covariate and treatment history up until that point.^22,31,32^

Pooled logistic regression was used to both generate the IPCW and to model time to death.^33,34,35^ In all analyses, time was discretized into days to ensure that the model would approximate an HR.^33,34,35^ Time was modeled using a restricted cubic spline with knots placed at the 10th, 50th, and 90th percentiles.^36^ Indicator variables were used for categorical variables, and linearity was assumed with respect to continuous covariates. No interaction terms were included in the model. Time to nonadherence was modeled as a function of time, the baseline covariates, and the time-varying covariates. The conditional probability estimates from this model were used to construct the IPCWs. The IPCWs were truncated at the 99th percentile and were not stabilized. Subsequently, a weighted pooled logistic regression was used to model time to death as a function of treatment strategy and time. To align with the IDEA trial analyses, we estimated an HR that was assumed common over time. Robust variance estimation was used to construct the 95% CIs. Subgroup analyses were performed by fitting separate outcome models within each subgroup of interest.

#### Missing Data and Loss to Follow-up

Patients missing baseline covariate information were excluded from the analyses. The last observation was carried forward when the patient was intermittently missing dose information for capecitabine, 5-flourourcail (bolus or intravenous), or leucovorin. Given our reliance on vital statistics to assess overall survival, we assumed that censoring would be noninformative.

#### Naive Analysis

Results from the trial emulation were compared with those from a naive observational analysis that did not attempt to explicitly emulate a hypothetical target trial. In particular, we generated a time-fixed treatment indicator variable whereby the value was determined according to the number of cycles actually completed by the patient. It should be noted that the assignment of patients to treatment groups based on the duration of therapy actually completed, as done in prior observational studies, is susceptible to immortal time bias.^25,32^ We similarly estimated a proportional HR using a pooled logistic regression model and adjusted for the same set of baseline covariates used in the trial emulation. The start of follow-up was similarly defined as start of treatment initiation. In contrast to the trial emulation, the routine observational analysis did not generate copies of the participants, artificially censor individuals at the time of nonadherence, or account for time-varying confounding. Therefore, the main differences between the naive observational analysis and trial emulation are that the former did not (1) account for allowable reasons for early cessation of therapy or treatment modifications related to cycle dosing and delays; (2) adjust for time-varying confounding; or (3) address immortal time bias.

#### Sensitivity Analysis

To further assess the generalizability of the IDEA trial findings to real-world populations, analyses were repeated after relaxing the inclusion criteria. Specifically, we made no exclusions based on time spent in the hospital in the past year or on comorbidities, and we relaxed the maximum duration of time from surgery to initiation of chemotherapy from 10 to 16 weeks.

## Results

**Figure 1.**
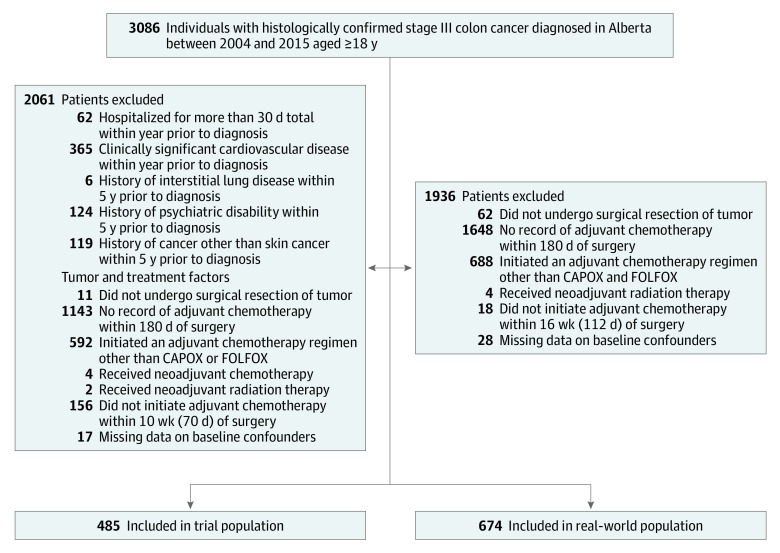
Flow Diagram Describing the Inclusion and Exclusion of Participants Within the Target Trial and Real-World Populations CAPOX indicates capecitabine plus oxaliplatin; FOLFOX, 5-fluorouracil/leucovorin plus oxaliplatin.

From an initial 3086 patients, 485 patients (16%) were included in our trial emulation (Figure 1). The median age was 59 years (range, 19-81 years), and 230 (47%) were women (Table 2). The maximum follow-up was 11.6 years. There were 90 deaths in the cohort. Median overall survival was not reached. The 5-year Kaplan-Meier overall survival estimate was 0.79 (95% CI, 0.75-0.84). In total, 281 of 485 individuals (58%) had data consistent with the 6-month treatment strategy, whereas 78 (16%) had data consistent with the 3- to 5-month treatment strategy.

**Table 2.** Baseline Characteristics of Study Populations Used in the Target Trial and Real-World Analyses

Population	Patients, No. (%)			
	Randomized trial, IDEA trial^4,26^ (n = 12 834)^a^	Observational study		
		Trial (n = 485)		Real world (n = 674)
Age, median (range), y	64 (18-88)	59.43 (18.50-81.19)	60.35 (18.50-86.53)	
Women	5590 (43.6)	230 (47.4)	319 (47.3)	
Men	7244 (56.4)	255 (52.6)	355 (52.7)	
BMI, mean (SD)	NA	28.09 (5.52)	28.08 (5.58)	
Charlson Comorbidity Index				
0	NA	358 (73.8)	425 (63.1)	
1	NA	79 (16.3)	111 (16.5)	
≥2	NA	48 (9.9)	138 (20.5)	
Time spent in hospital in year prior to diagnosis, mean (SD), d	NA	1.12 (2.92)	1.73 (9.44)	
Time from surgery to chemotherapy initiation, median (IQR), wk	NA	8.00 (6.86-8.86)	8.57 (7.29-9.86)	
Distance from home to treatment facility, median (IQR), km	NA	15.86 (9.19-32.64)	15.73 (8.80-32.44)	
Rural residence	NA	91 (18.8)	123 (18.2)	
T4 or N2 stage	5256 (41.3)	244 (50.3)	344 (51.0)	
Right-sided tumor	NA	250 (51.5)	358 (53.1)	
Low tumor grade	5399 (86.1)	379 (78.1)	530 (78.6)	
Treatment facility location				
Calgary	NA	155 (32.0)	218 (32.3)	
Edmonton	NA	269 (55.5)	376 (55.8)	
Regional or community	NA	61 (12.6)	80 (11.9)	
Year of diagnosis				
2005-2008	NA	49 (10.6)	72 (11.2)	
2009-2012	NA	213 (46.0)	297 (46.3)	
2013-2015	NA	201 (43.4)	273 (42.5)	
Prescribed FOLFOX	7763 (60.5)	316 (65.2)	453 (67.2)	

Abbreviations: BMI, body mass index (calculated as weight in kilograms divided by height in meters squared); IDEA, International Duration Evaluation of Adjuvant; IQR, interquartile range; NA, not applicable.

^a^ Percentages are based on those with available data.

**Figure 2.**
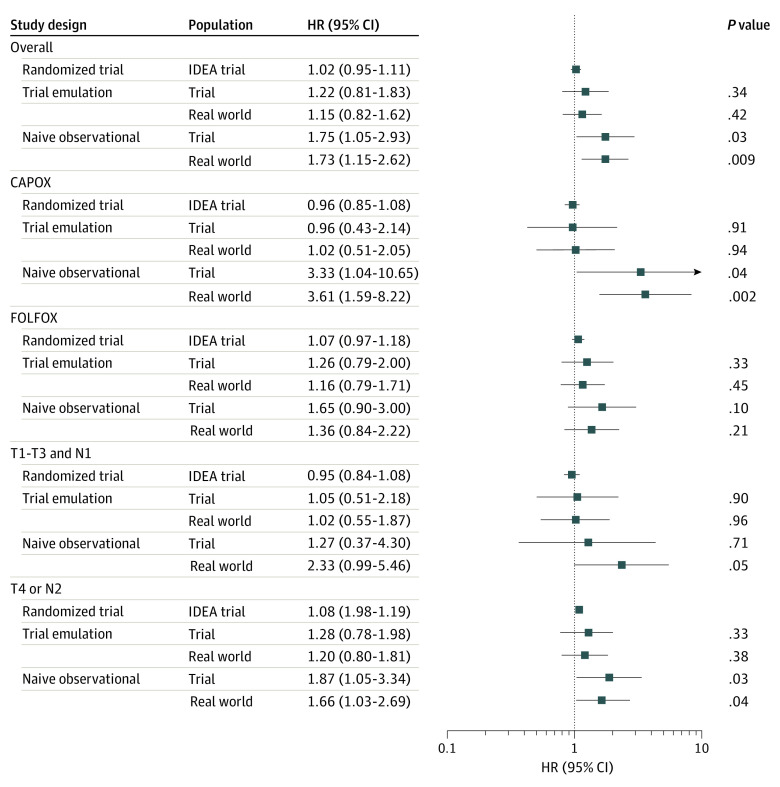
Overall Survival Estimates From Target Trial Emulation and Naive Observational Analysis With Those of the International Duration Evaluation of Adjuvant (IDEA) Trial HR indicates hazard ratio.

In general, the per-protocol estimates from the trial emulation were consistent with the intention-to-treat estimates from the IDEA trial for overall survival (Figure 2).^4,26^ In contrast, the naive analysis produced effect size estimates that were meaningfully different. For example, a shortened duration of adjuvant chemotherapy was found to be noninferior for overall survival among patients prescribed CAPOX in the IDEA trial (HR, 0.96; 95% CI, 0.85-1.08).^4,26^ Similar point estimates were obtained in our trial emulation (HR, 0.96; 95% CI, 0.43-2.14). However, in the naive analysis, a shortened duration of adjuvant chemotherapy was associated with decreased overall survival for patients prescribed CAPOX (HR, 3.33; 95% CI, 1.04-10.65).

In a sensitivity analysis, expansion of the eligibility criteria led to the inclusion of an additional 189 individuals, for a total sample size of 674 patients (Figure 1; Table 2). The findings from both the trial emulation and naive analyses were generally similar to those conducted within the trial population (Figure 2).

## Discussion

In a trial emulation, we obtained estimates that were consistent with those from the IDEA trial. Given our focus on the per-protocol treatment results and the use of real-world data, these findings may help to address some of the controversy surrounding the IDEA trial.^3,7,8^ The naive observational analysis that was not based on the explicit emulation of a target trial led to contradictory findings. Of particular note, the naive observational analysis suggested that a shortened duration of adjuvant CAPOX chemotherapy was associated with decreased overall survival, which contradicts the findings from the IDEA trial and current best practice guidelines.^3,4,5,6,7^ These findings add to the growing body of research highlighting the importance of explicitly emulating a target trial when conducting comparative efficacy research using real-world data, particularly with respect to the prevention of immortal time-bias.^15,19,23^

Within the methods section, 3 differences between the routine observational and target trial analyses were highlighted. To help identify the potential source for the disparate findings, 3 sensitivity analyses were conducted for the primary analysis, as follows: (1) we redefined the treatment strategy in terms of the actual duration of chemotherapy completed; (2) we repeated the trial emulation but did not adjust for time-varying confounding; and (3) we made both previously mentioned adjustments. These sensitivity analyses did not fully explain the disparity between the results from the trial emulation and naive analyses, particularly with respect to the CAPOX subgroup. As such, immortal time bias is likely the main reason for the disparate findings.

### Limitations and Strengths

There were a number of limitations with our trial emulation. First, our analyses lacked statistical precision due to our relatively small sample size (eg, there were 12 834 participants within the IDEA trial). Second, we relied on hospitalization and ambulatory records to assess treatment-related toxic effects. As such, we were unable to assess the grade and duration of the toxic effects and failed to capture low-grade toxic effects that did not result in hospitalization, which may have led to residual confounding. Because we were unable to assess the duration of toxic effects, we also may have misclassified some patients as being nonadherent to the treatment protocol given that patients in the IDEA trial were permitted to discontinue adjuvant chemotherapy if they did not recover from a grade 3 or higher toxic effect within 4 weeks.^4^ Third, we lacked information on Eastern Cooperative Oncology Group (ECOG) score and resection margin. While we attempted to use the number of a days spent in hospital within the year prior to diagnosis as a proxy for ECOG score, the surrogacy of this variable has not been validated. As a result, our analyses may have suffered from unmeasured confounding and our trial population may have differed from that of the IDEA trial. Fourth, we examined a treatment strategy that did not align exactly with the treatment strategy used in the IDEA trial (ie, we assessed 3 to 5 months instead of 3 months of adjuvant chemotherapy). Fifth, the IDEA trial did not provide estimates of the per-protocol effect size that accounted for postrandomization confounding.^10,37^ Because the intention-to-treat effect was not estimable within our observational data set, the per-protocol estimates from our trial emulation were compared with the intention-to-treat estimates from the IDEA trial. Disparity between the study populations, treatment strategies, and type of contrast (ie, per-protocol vs intention-to-treat) may explain the slight differences between our trial emulation and the IDEA trial with respect to the FOLFOX and T4 or N2 subgroup estimates.

Despite these limitations, there are notable strengths of our study. First, the design of our study was based on the explicit emulation of a target trial, which has been shown to mitigate a number of biases that often arise in observational research.^15,19,23^ Second, our statistical analyses adjusted time-varying confounding due to toxic effects, allowed for discontinuation due to cardiac toxicity, and accounted for the fact that the duration of treatment is unknown at baseline, unlike previous observational research to date.^25^ Third, our investigation relied on a large, population-based data set in which the overall survival was assessed through the use of vital statistics. As such, our results are likely generalizable to the broader Canadian population, and the risk of bias due to informative loss to follow-up is small.

## Conclusions

In this study, a target trial emulation investigating a shortened duration of adjuvant chemotherapy among individuals with stage III colon cancer obtained estimates that were similar to those from the IDEA trial using real-world data. In a naive observational analysis, the resulting estimates led to conclusions that conflicted with those from the IDEA trial. These analyses support the findings from the IDEA trial and highlight the importance of explicitly emulating a target trial when conducting observational analyses.

## Notes

Supplement. **eTable 1. **Description of *ICD-10-CM* Codes Used to Define Comorbid Conditions Used to Assess Patient Eligibility **eTable 2. **Description of *ICD-10-CM* Codes Used to Define Treatment Related Toxic Effects **eFigure. **Directed Acyclic Graph Describing the Assumed Structure of Time-Varying Confounding of the Number of Cycles of Adjuvant Chemotherapy and Overall Survival by Treatment-Related Toxic Effects Shown for the First 2 Chemotherapy Cycles **eReferences.**
