# Supplementary material for: Association of a Shortened Duration of Adjuvant Chemotherapy With Overall Survival Among Individuals With Stage III Colon Cancer
Source: JAMA Netw Open. 2021 Mar 30;4(3):e213587. doi: 10.1001/jamanetworkopen.2021.3587 (PMC8010592; doi:10.1001/jamanetworkopen.2021.3587)
Supplement: Supplement. — eTable 1. Description of ICD-10-CM Codes Used to Define Comorbid Conditions Used to Assess Patient Eligibility eTable 2. Description of ICD-10-CM Codes Used to Define Treatment Related Toxic Effects eFigure. Directed Acyclic Graph Describing the Assumed Structure of Time-Varying Confounding of the Number of Cycles of Adjuvant Chemotherapy and Overall Survival by Treatment-Related Toxic Effects Shown for the First 2 Chemotherapy Cycles eReferences. [file jamanetwopen-e213587-s001.pdf]

# Supplemental Online Content

Boyne DJ, Cheung WY, Hilsden RJ, et al. Association of a shortened duration of adjuvant chemotherapy with overall survival among individuals with stage III colon cancer. *JAMA Netw Open*. 2021;4(3):e213587. doi:10.1001/jamanetworkopen.2021.3587

**eTable 1.** Description of *ICD-10-CM* Codes Used to Define Comorbid Conditions Used to Assess Patient Eligibility
**eTable 2.** Description of *ICD-10-CM* Codes Used to Define Treatment Related Toxic Effects
**eFigure.** Directed Acyclic Graph Describing the Assumed Structure of Time-Varying Confounding of the Number of Cycles of Adjuvant Chemotherapy and Overall Survival by Treatment-Related Toxic Effects Shown for the First 2 Chemotherapy Cycles
**eReferences.**

This supplemental material has been provided by the authors to give readers additional information about their work.

© 2021 Boyne DJ et al. *JAMA Network Open.*

eTable 1. Description of *ICD-10-CM* Codes Used to Define Comorbid Conditions Used to Assess Patient Eligibility

| Construct | Condition | ICD-10-CM Code | Reference |
|---|---|---|---|
| Cardiovascular disease | Cerebrovascular disease | G45, G46, I60-I69, H340 | Quan (2005)[1] |
| | Myocardial infarction | I21, I22, I252 | Quan (2005)[1] |
| | Unstable angina | I200 | Henderson (2006)[2] |
| | Heart failure | I43, I50, I099, I110, I130, I132, I255, I420, I425-9, P290 | Quan (2005)[1] |
| | Cardiac dysrhythmias | I441-I443, I456, I459, I46-I49, R000, R001, R008, T821, Z450, Z950 | So (2006)[3] |
| | Uncontrolled hypertension | I10, I11, I12, I13, I15 | Henderson (2006)[2] |
| | Ischemic cardiopathy | I255 | - |
| Interstitial lung disease | Interstitial lung disease | J84 | - |
| | Pneumonitis | R06.02 | Pal (2019)[4] |
| Psychiatric disability | Dementia / Alzheimer's | F00-F03, G30, F051, G311 | Quan (2005)[1] |
| | Schizophrenia | F20, F22, F25, F30-39 | Jakobson (2005)[5] |
| | Intellectual disability | F70-79 | - |

© 2021 Boyne DJ et al. *JAMA Network Open.*

eTable 2. Description of *ICD-10-CM* Codes Used to Define Treatment Related Toxic Effects

| **Treatment-related toxicity** | **ICD-10-CM Code** |
|---|---|
| *A. Toxicities leading to an adherent discontinuation* | |
| Cardiovascular disease[a] | I20.X-I79.X, G08, G45, K55.X, R00.X, R01.X, R55.X, R56.X |
| Mental illness | F01.X-F09.X, F20.X-F33.X |
| Acute pancreatitis | K85.X |
| Kidney Failure | N17.X-N19.X |
| *B. Other treatment-related toxicities* | |
| Intestinal infectious diseases | A00.X-A09.X |
| Sepsis | A40.X, A41.X, A49.X, U82.X, U83.X |
| Hematological disease | D60.X-D64.X, D70.X, D72.X |
| Dehydration | E86.X-E87.X |
| Hypertensive diseases | I05.X-I09.X, I95.X |
| Thrombosis | I81.X, I82.X |
| Respiratory toxicity | J00.X-J99.X, R06.X |
| Hematuria | R31.X |
| Fever | R50.X |
| Nausea and vomiting | R11.X |
| Urinary tract infection | N39.0 |
| Neuropathy | G62.0, G20.09, R20 |
| Colorectal fissure, fistula, or abscess | K60.X, K61.X |
| Post-surgical infection | T81.4X, T82.6X-T82.8X |
| Gastrointestinal ulcer | K25.X-K28.X, K62.6, K63.3 |
| Stomatitis, gastritis, cholecystitis, and peritonitis | K12.X, K29.X, K65.X, K81.X |
| Enteritis and colitis | K50.X-K52.X |
| Acute lymphangitis | L03.X |
| Obstruction and diverticular disease | K56.X, K57.X, K63.0, K63.2, K91.3 |
| Other complications of antineoplastic drugs | Y43.3 |

[a] Includes: ischemic, pulmonary and other forms of heart disease as well as cerebrovascular disease and diseases of arteries, arterioles, and capillaries; Excludes: hypertensive, rheumatic diseases, and other diseases of the veins, lymphatic vessels and lymph node

© 2021 Boyne DJ et al. *JAMA Network Open.*

**eFigure. Directed Acyclic Graph Describing the Assumed Structure of Time-Varying Confounding of the Number of Cycles of Adjuvant Chemotherapy and Overall Survival by Treatment-Related Toxic Effects Shown for the First 2 Chemotherapy Cycles**

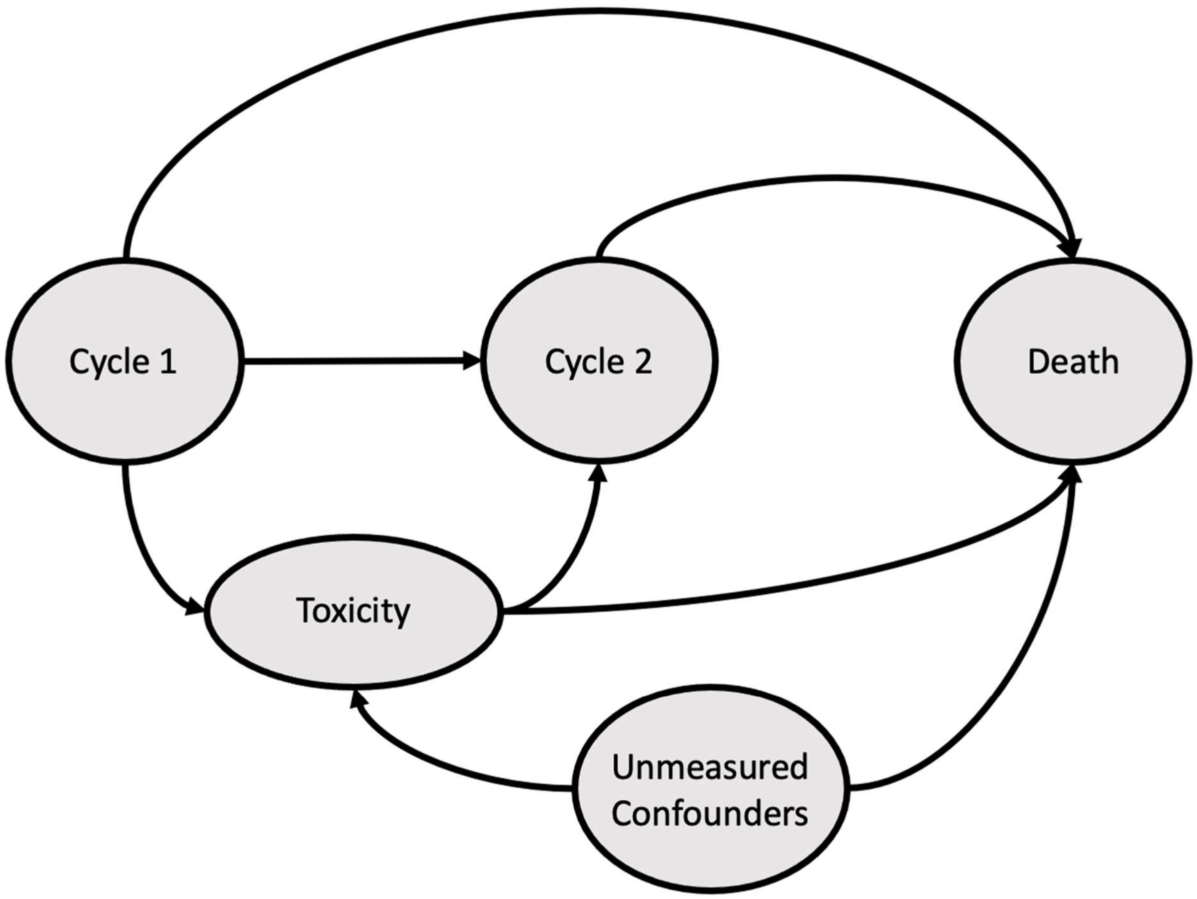


© 2021 Boyne DJ et al. *JAMA Network Open.*

**eReferences**


1. Quan H, Sundararajan V, Halfon P, et al. Coding algorithms for defining comorbidities in ICD-9-CM and ICD-10 administrative data. Medical care. 2005;43(11):1130-1139.
2. Henderson T, Shepheard J, Sundararajan V. Quality of diagnosis and procedure coding in ICD-10 administrative data. Medical care. 2006;44(11):1011-1019.
3. So L, Evans D, Quan H. ICD-10 coding algorithms for defining comorbidities of acute myocardial infarction. BMC Health Serv Res. 2006;6:161.
4. Pal S, Gong J, Mhatre SK, et al. Real-world treatment patterns and adverse events in metastatic renal cell carcinoma from a large US claims database. BMC Cancer. 2019;19(1):548.
5. Jakobsen KD, Frederiksen JN, Hansen T, Jansson LB, Parnas J, Werge T. Reliability of clinical ICD-10 schizophrenia diagnoses. Nord J Psychiatry. 2005;59(3):209-212.


© 2021 Boyne DJ et al. *JAMA Network Open.*
